# Sugar-sweetened beverages and colorectal cancer risk in the California Teachers Study

**DOI:** 10.1371/journal.pone.0223638

**Published:** 2019-10-09

**Authors:** Lorena S. Pacheco, Cheryl A. M. Anderson, James V. Lacey, Edward L. Giovannucci, Hector Lemus, Maria Rosario G. Araneta, Dorothy D. Sears, Gregory A. Talavera, Maria Elena Martinez

**Affiliations:** 1 Department of Family Medicine and Public Health, School of Medicine, University of California San Diego, La Jolla, California, United States of America; 2 School of Public Health, San Diego State University, San Diego, California, United States of America; 3 Division of Health Analytics, Department of Computational and Quantitative Medicine, City of Hope, Duarte, California, United States of America; 4 Department of Nutrition, Harvard T.H. Chan School of Public Health, Boston, Massachusetts, United States of America; 5 Department of Epidemiology, Harvard T.H. Chan School of Public Health, Boston, Massachusetts, United States of America; 6 College of Health Solution, Arizona State University, Phoenix, Arizona, United States of America; 7 Moores Cancer Center, University of California San Diego, La Jolla, California, United States of America; National Cancer Institute, UNITED STATES

## Abstract

**Background:**

The association between sugar-sweetened beverage (SSB) consumption and colorectal cancer (CRC) risk remains unclear and published data are limited.

**Methods:**

The analytic cohort included 99,798 women, free of cancer at baseline, from the California Teachers Study, a longitudinal cohort comprised of 133,477 female teachers and administrators who were active or recently retired members of the California State Teachers Retirement System in 1995. SSB consumption constituted caloric soft drinks, sweetened bottled waters and teas, and fruit drinks, derived from a self-administered food frequency questionnaire. Consumption was divided into four categories: Rare or never, >rare/never to <1 serving/week, ≥1 serving/week to <1 serving/day, and ≥1 serving/day. CRC endpoints were based on annual linkage with California Cancer Registry, defined as first diagnosis of CRC, and classified following the Surveillance, Epidemiology, and End Results Program coding system. Multivariable-adjusted Cox proportional hazards models were used to generate hazard ratios (HR) and 95% confidence intervals (CI) for assessing the association between SSB consumption and incident CRC.

**Results:**

A total of 1,318 incident CRC cases were identified over 20 years of follow-up (54.5% proximal colon and 45.5% distal colorectum). Compared with rare/never consumers, the multivariable-adjusted HRs (95% CI) were 1.14 (0.86, 1.53) for total CRC; 1.11 (0.73, 1.68) for proximal colon; and 1.22 (0.80, 1.86) for distal colorectum cancers among women consuming ≥ 1 serving/day of SSBs.

**Conclusion:**

SSBs were not significantly associated with CRC risk. The biological effects of high SSB consumption make it important to continue to evaluate whether SSBs are associated with CRC. Additionally, future studies should further assess SSBs in large, racial/ethnically diverse cohorts of males and females, and, if feasible, address changes in SSB consumption over time.

## Introduction

Globally, colorectal cancer (CRC) is the third most commonly diagnosed cancer in adult men and the second most commonly diagnosed cancer in women [1]. In the United States (U.S.), CRC is the third most frequently occurring malignancy in both adult men and women [2]. Incidence and death rates vary according to nation-specific developmental and economic levels, alluding to the influence of environmental and lifestyle factors, in the development of CRC [1], [3]. Specifically, physical inactivity and sedentary behavior, excessive caloric intake, obesity, and a Westernized dietary pattern, are lifestyle factors that are associated with an increased risk of CRC [4]–[9].

There are notable epidemiological and pathophysiological sex differences in CRC features, including tumor location and CRC subtype [10]–[13]. Although women have lower CRC incidence and mortality rates compared to men [14], they have a higher risk of developing proximal colon cancer, which is characterized by microsatellite instability stemming from impaired gene mismatch repair activity and CpG (cytosine nucleotide followed by a guanine nucleotide) methylation [15], [16]. On the other hand, men are more likely to develop distal colorectum cancer, featuring chromosomal instability with downregulation of tumor suppressor genes and upregulation of oncogenes [15]–[17]. Proximal colon cancer often presents with a more advanced stage at diagnosis and tends to be more aggressive than distal colorectum cancer [13], [18].

Consumption of sugar-sweetened beverages (SSBs), a component of a Westernized dietary pattern, has increased worldwide [19]–[21]. SSBs are manufactured carbonated and noncarbonated beverages containing caloric sweeteners or syrups (i.e. high-fructose corn syrup) and include, but not limited to, regular soft drinks (not sugar-free), fruit drinks, sports and energy drinks, sweetened waters, and tea and coffee beverages with added sugars [22]. A comprehensive 187-country analysis reported higher per capita SSB consumption in upper-middle vs. lower-middle income countries [23]. Average SSB consumption among U.S. adults was 1.0 serving/day, corresponding to 26th-highest intake of SSBs [23].

There is substantial evidence that frequent and/or excessive consumption of SSBs leads to weight gain, general and central obesity, and type 2 diabetes [5], [24]. This is particularly important since CRC is one of the 13 obesity-related cancers [25]. The Continuous Update Project (CUP), combined effort of the World Cancer Fund and American Cancer Research Institute, concluded that there is strong, convincing evidence of higher body fatness and increased risk of CRC, recommending a healthy weight for risk reduction [1]. Additionally, the CUP recommends limiting the consumption of SSBs while promoting water or unsweetened beverages, with the ultimate goal of excluding SSBs from the diet. In spite of this recommendation, published studies specifically examining the relationship between SSB intake and risk of colon cancer [26]–[28] and CRC [29] are limited and inconsistent. A pooled analysis in 2010 reported a null association between sugar-sweetened carbonated soft drink consumption and colon cancer risk [30]. Results of a more recent prospective study showed sugar-sweetened soft drink consumption was positively associated with risk of CRC [29].

We examined the association between SSB consumption and incident CRC, including risk by CRC tumor location, in a large prospective cohort of middle-aged women. Our study contributes to the literature by providing data on SSB consumption, as a composite, as well as examining risk for total CRC and by subsite.

## Materials and methods

### Study population and design

The California Teachers Study (CTS) is an ongoing prospective cohort study comprised of 133,477 active and retired female teachers and administrators, who completed a 16-page mailed questionnaire at study enrollment in 1995–1996 and members of the California State Teachers Retirement System. Methodological details of the cohort have been previously published [31]. The baseline questionnaire encompassed a comprehensive range of participant information including demographic and lifestyle characteristics, behavioral factors, family history of chronic disease, medical history and co-morbidities. Annual follow-up captures change of residence, cancer diagnoses, hospitalizations, ambulatory care procedures, and deaths. Change of residence is attained by mailings and participant communication. Cancer diagnoses are ascertained by linkage with the California Cancer Registry. Linkage with the Office of Statewide Health Planning and Development provides hospitalization and ambulatory care procedures and diagnoses performed in California. Date and cause of death are determined using state and national mortality files and National Death Index.

The CTS study was approved by the Institutional Review Boards at the participating institutions. This analysis was approved by the Institutional Review Boards of City of Hope and the University of California San Diego.

### Dietary assessment and sugar-sweetened beverage intake

Dietary intake during the year preceding baseline was assessed using a validated 103-item self-administered food frequency questionnaire (FFQ), developed from a former version of the Block 95 FFQ. Usual serving size (i.e., small medium, large or extra-large serving) and frequency of consumption (i.e., never or <1 time/month, 1 time/month, 2–3 times/month, 1 time/week, 2 times/week, 3–4 times/week, 5–6 times/week, every day, and/or ≥2 times/day) of the 103 food and beverage items was characterized. The reproducibility and validity of this instrument in the cohort has been described elsewhere [32]. SSB consumption determination comprised a composite of sweetened carbonated and noncarbonated beverages, including caloric soft drinks, sweetened bottled waters and/or teas, and fruit drinks (other than fruit juice), derived from 3 items on the FFQ: ‘Regular soft drinks (not diet soda)’, ‘Snapple, Calistoga, sweetened bottled waters or iced teas’, and ‘Kool-Aid, Hi-C, or other drinks with added Vitamin C’. From the 9 possible frequency categories ranging from ‘never or less than once per month’ to ‘≥2 times/day’, SSB consumption was divided into four categories: Rare or never, >rare/never to <1 serving/week, ≥1 serving/week to <1 serving/day, and ≥1 serving/day. A serving of SSB consisted of 8 fluid ounces (fl oz), approximate weight 237 grams, for sweetened bottled water and/or teas and fruit drinks, and 12 fl oz, approximate weight 355 grams, for caloric soft drinks.

### Ascertainment of colorectal cancer

CRC incident cases were ascertained by linkage with the California Cancer Registry, a statewide population-based cancer registry where cancer diagnoses in California residents are reported that participates in the National Cancer Institute’s Surveillance, Epidemiology, and End Results (SEER) program. Annual linkage determined incident cancers diagnosed through December 31, 2015 among CTS participants. Incident CRC cases were operationally identified by SEER codes, with cancers located in the cecum, appendix, ascending colon, hepatic flexure, transverse colon, and splenic flexure (SEER codes 21041–21046) categorized as proximal colon and cancers located in the descending colon, sigmoid colon, large intestine, rectosigmoid junction, and rectum (SEER codes 21047–21049, 21051, and 21052) categorized as distal colorectum.

### Assessment of covariates

Demographic and lifestyle characteristics from the baseline questionnaire were considered as possible confounders. Covariates included age, race/ethnicity, socioeconomic status (SES), total years smoked, alcohol intake, family history of colorectum cancer, history of polyps, diabetes, moderate to vigorous physical activity (MVPA), aspirin frequency and duration, multivitamin frequency and duration, menopausal status and menopausal hormone therapy use, oral contraceptive use, body mass index (BMI), total energy intake, and a set of dietary intake factors.

SES was determined by combining three 1990 U.S. block census data variables (occupation, education, and family income); where all block groups in the state were ranked by occupation (% adults employed in managerial/professional occupation), level of education (% of adults over the age of 25 completing at least a college degree), and median family income, corresponding to quartiles analogous the statewide adult population. A summary score was developed for SES with categories ranging from 1 (lowest) to 4 (highest). Total years smoked was calculated based on age of first and last smoke for those participants who reported smoking at least 100 cigarettes in their lifetime. Alcohol intake was determined from frequency and number of drinks per week of beer, champagne and/or wine, and cocktails and/or liquor. Physical activity, including MVPA, was estimated using questionnaire-derived intensity, duration, and frequency of listed activities, on an average day. BMI (kg/m^2^) was calculated as weight (kg) divided by height squared (m^2^), from self-reported weight and height.

### Analytic sample

**Fig 1:**
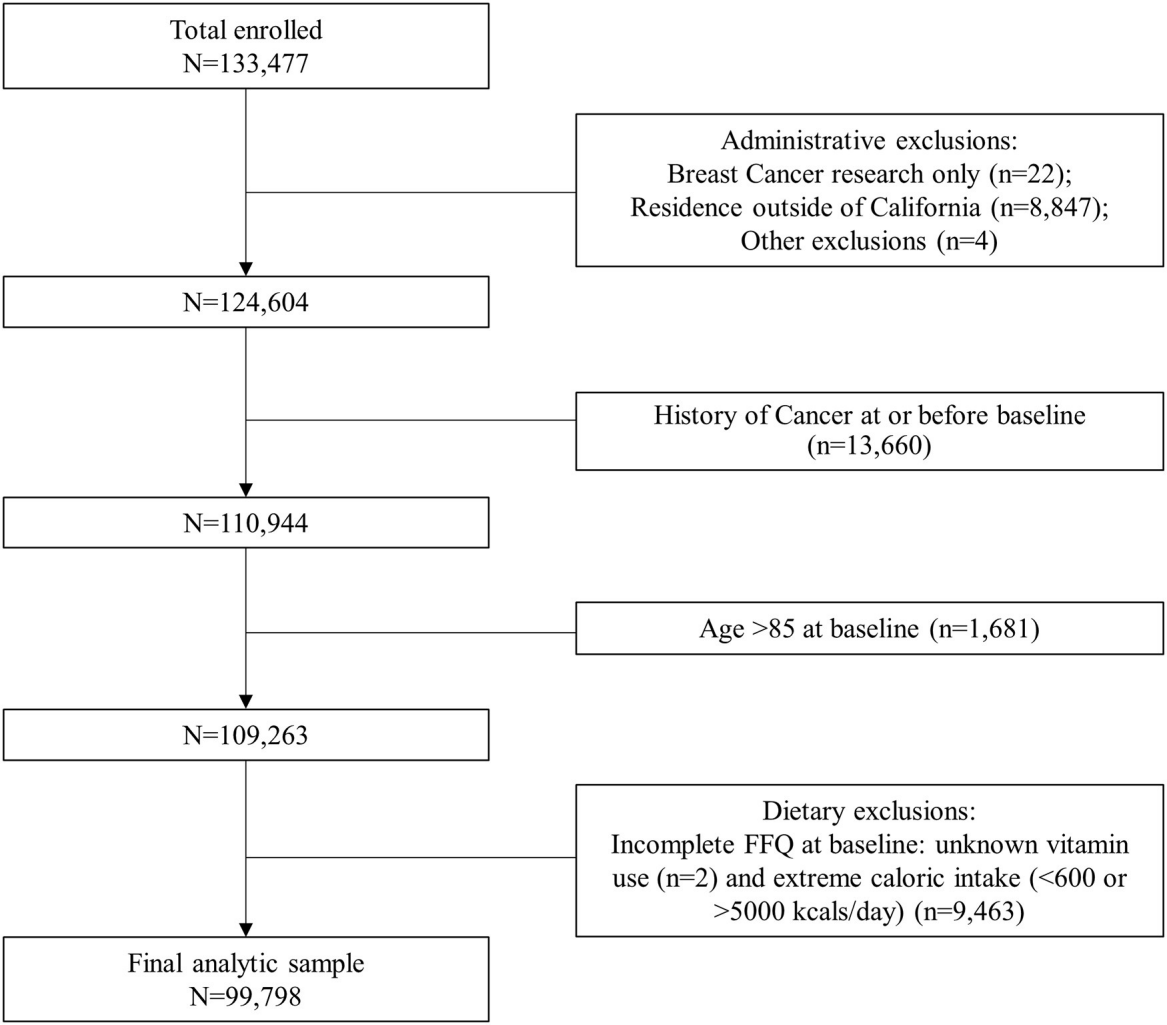
Flowchart of analytic sample for sugar-sweetened beverages and colorectal cancer risk in the CTS.

For the current analysis, we excluded participants who specified their data only be used for breast cancer research (n = 22), those who resided outside of California at baseline (n = 8,847), returned incomplete or incomprehensible questionnaires (n = 4), those with a history of cancer at baseline (n = 13,660), were age ≥ 85 years at baseline (n = 1,681), had extreme caloric intake values (<600 [n = 8,950] or >5000 kcal/day [n = 513]) or had incomplete FFQ data at baseline including vitamin use (n = 2), yielding a final analytic sample of 99,798 female participants for follow-up (Fig 1).

### Statistical analyses

Mean and standard error of mean (SEM) or proportion and frequency were calculated for baseline characteristics of cohort participants in each SSB consumption category. Cox proportional hazard modeling was used to estimate hazard ratios (HRs) and 95% confidence intervals (CI) of total CRC risk according to SSB consumption, and separately for proximal colon and distal colorectum cancers. The independent associations between type of SSB and incident CRC were also examined. Linear trend was modeled by assigning each participant the median intake in her respective SSB intake category and included as a continuous independent variable in the Cox proportional hazard model. The proportional hazards assumption was met by inspecting the survival curves according to SSB consumption categories as well as testing time-varying covariates in the model. Cohort members contributed person-years to the analysis from date of baseline questionnaire completion until first CRC diagnosis date, relocation out of California, death, or December 31, 2015, whichever occurred earliest.

For the multivariate analysis, we adjusted for the following potential confounders: age, race/ethnicity (White, Asian/Pacific Islander, African-American, Hispanic/Latino, Native-American, or Mixed/Other; further categorized as non-Hispanic White vs all other), SES (quartiles: 1^st^, 2^nd^, 3^rd^, 4^th^, or unknown), total years smoked, alcohol intake (0, <20, or ≥20 grams/day), family history of colon cancer (yes or no), history of polyps (yes or no), diabetes (yes or no), MVPA (quintiles minutes/week: 0–30, 30–97.8, 97.8–202.8, 202.8–360, >360, and unknown), aspirin use (1–3 times/week, 4–6 times/week, daily, regular use but undetermined frequency, or unknown), multivitamin use (never, 1–3 times/week, 4–6 times/week, daily, regular use but undetermined frequency), menopausal status and menopausal hormone therapy use (premenopausal, perimenopausal/postmenopausal with never, past, or current hormone therapy use of estrogen, estrogen + progesterone, or other hormone combinations), and oral contraceptive use (never, past or current). We further adjusted for the following possible mediators: BMI, total energy intake, and a set of dietary intake covariates: red meat, processed meat, and non-starchy vegetable. Intake of these three diet components were adjusted for total energy by using the residual method [33], before including them in the model. A total of three progressively adjusted multivariable Cox regression models were fitted after the age-adjusted model. Model 1 included all the above-mentioned covariates except for BMI, total energy intake, and dietary intake covariates. Model 2 additionally adjusted for BMI, total energy intake and intake of red and processed meat and non-starchy vegetables. The final model includes covariates that were known and tested (if ≥10% change in HR) confounders in this exposure and outcome association. Variables with a *P* value ≤0.05 remained in the final model. Additionally, the models examining the association between sweetened bottled waters and/or tea, fruit drink, and caloric soft drink consumption and risk of CRC, were reciprocally adjusted for the other beverage types (i.e. the sweetened water or tea analysis was adjusted for fruit drink and caloric soft drink, and vice versa).

Sensitivity analysis excluded CRC cases diagnosed within the first 2 and 4 years of follow-up. We also conducted analysis stratified by BMI (underweight, normal weight, overweight, and obese) given biological plausibility supporting stronger effect of SSB in overweight/obese individuals who would have insulin resistance [9], [34], [35]. All *P* values presented are 2-tailed; *P* < .05 was considered statistically significant. Analyses were conducted with SAS version 9.4 (SAS Institute Inc, Cary, NC).

## Results

CTS participants were, on average (mean ± standard deviation), aged 52.0 ± 13.5 years at baseline, and were followed for a median of 20.1 years, contributing 1,743,453 person-years. During follow-up, we ascertained 1,318 incident cases of CRC, of which 54.5% (n = 718) were proximal and 45.5% (n = 600) were distal colorectum cases. Table 1 reports baseline demographic and lifestyle characteristics for participants according to SSB consumption. Women who consumed ≥1 serving/day of SSBs (SSB daily consumers), which comprised 4.3% of all participants, had an average daily SSB intake of 13.5 ± 0.05 fl oz. These daily consumers tended to have higher intake of total energy, carbohydrate, red and processed meat, and lower intake of protein, fat, and fruit and vegetables compared to the rare/never consumers. They were also more likely to be current smokers (7.6%) with an average (mean ± SEM) total years smoked of 20.3 ± 0.35, past or current OC users (74.8%), premenopausal (47.9%), and had the highest obesity rates (18.2%). Comprehensive participant characteristics are reported in S1 Table.

**Table 1:** Baseline characteristics of California Teachers Study participants according to sugar-sweetened beverage consumption categories*.

Characteristic	Rare or never	>rare/never to <1 serving per week	≥1 serving per week to <1 serving per day	≥1 serving per day
**N**	40,911	33,198	21,403	4,286
**Age, y**	55.5 ± 0.06	48.9 ± 0.07	48.8 ± 0.09	48.8 ± 0.20
**Race/ethnicity, %**				
White	36,667 (89.6)	27,919 (84.1)	18,147 (84.8)	3,750 (87.5)
All other	4,244 (10.4)	5,279 (15.9)	3,256 (15.2)	536 (12.5)
**Education**^ǂ^, **%**				
Academic/Professional doctorate	1,012 (2.5)	719 (2.2)	494 (2.3)	120 (2.8)
Master’s degree	10,494 (25.7)	8,934 (26.9)	5,686 (26.6)	1,150 (26.8)
Bachelor’s degree	9,112 (22.3)	7,746 (23.3)	4,518 (21.1)	868 (20.3)
Associate’s degree or less	138 (0.3)	139 (0.4)	100 (0.5)	21 (0.5)
Unknown	20,155 (49.3)	15,660 (47.2)	10,605 (49.6)	2,217 (49.6)
**Occupation, %**				
Teacher, any kind	21,149 (51.7)	21,313 (64.2)	13,968 (65.3)	2,881 (67.2)
Pupil services	1,146 (2.8)	1,099 (3.3)	690 (3.2)	136 (3.2)
Administration	1,311 (3.2)	1,221 (3.7)	866 (4.1)	198 (4.6)
Any other combination	593 (1.5)	602 (1.8)	388 (1.8)	73 (1.7)
Unknown	16,712 (40.9)	8,963 (27.0)	5,491 (25.7)	998 (23.3)
**Socioeconomic status, %**				
1^st^ quartile, low	1,614 (4.0)	1,493 (4.5)	959 (4.5)	177 (4.1)
2^nd^ quartile, low-medium	6,728 (16.5)	5,846 (17.6)	3,835 (17.9)	718 (16.8)
3^rd^ quartile, medium-high	13,030 (31.9)	11,072 (33.4)	6,891 (32.2)	1,436 (33.5)
4^th^ quartile, high	19,017 (46.5)	14,328 (43.2)	9,435 (44.1)	1,904 (44.4)
Unknown	522 (1.3)	459 (1.4)	283 (1.3)	51 (1.2)
**Marital status, %**				
Married	18,457 (45.1)	16,268 (49.0)	10,016 (46.8)	1,973 (46.0)
Separated/Divorced	3,831 (9.4)	2,977 (9.0)	1,825 (8.5)	392 (9.2)
Widowed	3,346 (8.2)	1,558 (4.7)	999 (4.7)	178 (4.2)
All other	15,277 (37.3)	12,395 (37.3)	8,563 (40.0)	1,743 (40.7)
**Dietary Intake**				
Energy, kcal/day	1755.3 ± 3.35	1954.14± 3.72	2046.5 ± 4.64	2255.4 ± 10.36
Carbohydrate, g/day	251.5 ± 0.18	253.4 ± 0.20	260.2 ± 0.25	282.46± 0.55
Protein, g/day	80.6 ± 0.07	76.8 ± 0.07	74.4 ± 0.09	68.0 ± 0.20
Total Fat, g/day	60.0 ± 0.07	61.6 ± 0.07	59.8 ± 0.09	53.8 ± 0.20
Fruit and vegetable, g/day	359.7 ± 0.87	299.9 ± 0.96	285.6 ± 1.19	266.1 ± 2.67
Vegetables, g/day	183.8 ± 0.54	163.4 ± 0.60	163.4 ± 0.74	167.7 ± 1.67
Red meat, g/day	29.4 ± 0.17	34.7 ± 0.19	36.1 ± 0.23	37.3 ± 0.52
Processed meat intake, g/day	6.5 ± 0.06	8.2 ± 0.06	8.5 ± 0.08	8.9 ± 0.18
**SSB intake, fl oz**	0 ± 0.02	2.6 ± 0.02	5.5 ± 0.02	13.5 ± 0.05
**MVPA, minutes/week**	236.3 ± 1.22	213.7 ± 1.35	219.5 ± 1.68	220.7 ± 3.76
**Smoking, current, %**	2,084 (5.1)	1,494 (4.5)	1,100 (5.1)	321 (7.5)
**Total years smoked**, ^¥^	21.1 ± 0.11	17.8 ± 0.14	18.4 ± 0.17	20.0 ± 0.36
**Alcohol consumption, ≥20 g/day, %**	3,956 (9.7)	2,377 (7.2)	1,602 (7.5)	322 (7.5)
**Body mass index, kg/m**^**2**^	24.9 ± 0.03	24.6 ± 0.03	25.0 ± 0.04	25.7 ± 0.08
**Obese, body mass index ≥30 kg/m**^**2**^, **%**	5,487 (13.4)	4,217 (12.7)	3,079 (14.4)	787 (18.4)
**Hypertension, %**	7,842 (19.2)	4,288 (12.9)	3,022 (14.1)	673 (15.7)
**Diabetes, %**	1,712 (4.2)	434 (1.3)	344 (1.6)	109 (2.5)
**Daily aspirin use, %**	3,656 (8.9)	1,736 (5.2)	1,231 (5.8)	294 (6.9)
**Daily antihypertensive medication use, %**	7,201 (17.6)	3,679 (11.1)	2,622 (12.3)	599 (14.0)
**Daily multivitamin use, %**	16,355 (40.0)	10,578 (31.9)	6,930 (32.4)	1,492 (34.8)
**Cancer family history**^¤^, **%**	22,250 (54.4)	17,160 (51.7)	11,083 (51.8)	2,257 (52.7)
**Colorectum cancer family history**^§^, **%**	3,791 (9.3)	2,562 (7.7)	1,711 (8.0)	312 (7.3)
**Menopausal status and menopausal HT use, %**				
Premenopausal	13,084 (32.0)	16,777 (50.5)	10,722 (50.1)	2,098 (49.0)
PP, no HT	5,639 (13.8)	2,879 (8.7)	1,913 (8.9)	380 (8.9)
PP, past HT	3,241 (7.9)	1,620 (4.9)	1,038 (4.9)	214 (5.0)
PP, current HT Estrogen	6,287 (15.4)	3,567 (10.7)	2,257 (10.6)	480 (11.2)
PP, current HT Estrogen & Progesterone	7,128 (17.4)	4,335 (13.1)	2,739 (12.8)	510 (11.9)
All other	5,532 (13.5)	4,020 (12.1)	2,734 (12.8)	604 (14.1)
**Oral contraceptive use, past and current, %**	24,524 (62.3)	23,629 (73.4)	15,380 (74.3)	3,097 (75.1)

*Values are n (%) for categorical variables and means ± standard error of means for continuous variables.

^ǂ^Education was obtained after baseline, during fourth mail-in questionnaire follow-up, 2005–2006, where a total of n = 67,789 participants completed the questionnaire.

^¥^Former or current smokers.

^¤^ Cancer family history includes breast, endometrial, ovarian, cervical, lung, thyroid, colon, rectal, prostate, melanoma, and skin cancers, and also leukemia, and Hodgkin’s lymphoma history, of first-degree relatives (parent, sibling, offspring).

^§^Colorectum cancer family history includes disease in first-degree relatives (parent, sibling, offspring); fl oz, fluid ounces; g/day, grams per day; HT, hormone therapy; kcal/day, kilocalories per day; mo, months; MVPA, moderate-vigorous physical activity; PP, peri- or post-menopausal; SSB, sugar-sweetened beverage; y, years.

The HR (95% CI) for total CRC risk and SSB consumption was 1.14 (0.86, 1.53) comparing women who were SSBs daily consumers versus those who rarely/never consumed SSBs (Final model, Table 2). The HR (95% CI) for proximal colon cancer was 1.07 (0.71, 1.62) and that for distal colorectum cancer was 1.22 (0.82, 1.83) in the final multivariable-adjusted model (all *P* trend >0.05). There was no significant association between SSB intake and total CRC after taking into account potential confounders including CRC risk factors (Model 1), BMI, and dietary intake (Model 2) (Table 2). In regards to type of SSB and CRC, women who consumed ≥1 serving/day of sweetened bottled water and/or tea had a HR (95% CI) for total CRC risk of 1.21 (0.91, 1.60), compared to those who were rare/never consumers (Table 3). Caloric soft drink consumption was not associated with total CRC risk; women who consumed ≥1 serving/day of caloric soft drink had a HR (95% CI) for total CRC risk of 0.98 (0.65, 1.46), compared to those who were rare/never consumers (Table 3).

**Table 2:** Colorectal cancer risk* according to sugar-sweetened beverage consumption.

	Sugar-Sweetened Beverage Consumption ^†^				
Colorectal Cancer	Rare or never	>rare/never to <1 serving per week	≥1 serving per week to <1 serving per day	≥1 serving per day	P trend
**Total**					
No. of cases	663	354	247	54	
Rate per 10,000 person-years	9.5	6.0	6.5	7.1	
Age-adjusted HR (95% CI)	1.0	0.91 (0.80, 1.04)	1.02 (0.88, 1.18)	1.16 (0.88, 1.53)	
Multivariable-adjusted HR (95% CI)					
Model 1^ǂ^	1.0	0.92 (0.80, 1.05)	1.01 (0.87, 1.18)	1.15 (0.87, 1.52)	
Model 2^¥^	1.0	0.91 (0.80, 1.05)	1.04 (0.89, 1.21)	1.16 (0.87, 1.54)	
Final Model^¤^	1.0	0.92 (0.80, 1.05)	1.04 (0.89, 1.21)	1.14 (0.86, 1.53)	0.259
**Proximal Colon**					
No. of cases	375	197	120	26	
Rate per 10,000 person-years	5.4	3.7	5.2	3.4	
Age-adjusted HR (95% CI)	1.0	0.97 (0.81, 1.16)	0.94 (0.77, 1.16)	1.08 (0.72, 1.60)	
Multivariable-adjusted HR (95% CI)					
Model 1^ǂ^	1.0	0.97 (0.81, 1.15)	0.93 (0.76, 1.15)	1.08 (0.72, 1.61)	
Model 2^¥^	1.0	0.98 (0.81, 1.18)	0.96 (0.78, 1.20)	1.09 (0.72, 1.65)	
Final Model^¤^	1.0	0.96 (0.80, 1.15)	0.94 (0.76, 1.17)	1.07 (0.71, 1.62)	0.998
**Distal Colorectum**					
No. of cases	288	157	127	28	
Rate per 10,000 person-years	4.1	2.7	3.4	3.7	
Age-adjusted HR (95% CI)	1.0	0.86 (0.71, 1.05)	1.12 (0.90, 1.38)	1.25 (0.85, 1.85)	
Multivariable-adjusted HR (95% CI)					
Model 1^ǂ^	1.0	0.85 (0.70, 1.04)	1.09 (0.88, 1.35)	1.22 (0.82, 1.80)	
Model 2^¥^	1.0	0.85 (0.68, 1.04)	1.11 (0.89, 1.39)	1.22 (0.82, 1.84)	
Final Model^¤^	1.0	0.87 (0.71, 1.07)	1.14 (0.92, 1.42)	1.22 (0.82, 1.83)	0.101

*Total person-time: 1,743.453 years.

^†^ 1 serving of caloric soft drink is 12 fluid ounces, 1 serving of sweetened bottled water/tea or fruit drink is 8 fluid ounces. HR, hazard ratio; CI, confidence interval.

^ǂ^Model 1 adjusted for: age, race/ethnicity, socioeconomic status, total years smoked, alcohol intake, colorectum cancer family history of first-degree relatives, history of polyps, diabetes, physical activity, aspirin use, multivitamin use, menopausal status, menopausal hormone therapy use, oral contraceptive use.

^¥^Model 2 adjusted for: Model 1 and body mass index, total energy intake, and dietary variables: red meat, processed meat, and vegetable intakes.

^¤^Final model adjusted for: age, total years smoked, alcohol intake, colorectum cancer family history of first-degree relatives, history of polyps, multivitamin use, menopausal status, menopausal hormone therapy use, body mass index, and total energy intake.

**Table 3:** Colorectal cancer risk* according to specific sugar-sweetened beverage consumption.

	Sugar-Sweetened Beverage Consumption †				
Total Colorectal Cancer	Rare or never	>rare/never to <1 serving per week	≥1 serving per week to <1 serving per day	≥1 serving per day	P trend
	**Sweetened bottled water and/or tea**				
No. of cases	876	201	185	56	
Rate per 10,000 person-years	8.8	5.7	5.7	7.2	
Age-adjusted HR (95% CI)	1.0	0.82 (0.82, 1.13)	0.97 (0.83, 1.15)	1.23 (0.94, 1.61)	
Multivariable-adjusted HR (95% CI)					
Model 1^ǂ^	1.0	0.98 (0.84, 1.15)	0.99 (0.84, 1.17)	1.24 (0.94, 1.62)	
Model 2^¥^	1.0	0.98 (0.83, 1.15)	0.99 (0.84, 1.17)	1.24 (0.93, 1.64)	
Final model^¤^	1.0	0.97 (0.82, 1.14)	0.98 (0.83, 1.16)	1.21 (0.91, 1.60)	0.287
	**Fruit drinks**				
No. of cases	1,233	53	30	2	
Rate per 10,000 person-years	7.9	5.1	4.6	2.5	
Age-adjusted HR (95% CI)	1.0	0.97 (0.73, 1.28)	0.89 (0.62, 1.28)	0.45 (0.11, 1.81)	
Multivariable-adjusted HR (95% CI)					
Model 1^ǂ^	1.0	0.95 (0.72, 1.26)	0.87 (0.60, 1.25)	0.44 (0.11, 1.77)	
Model 2^¥^	1.0	0.91 (0.68, 1.21)	0.91 (0.63, 1.32)	0.48 (0.12, 1.91)	
Final model^¤^	1.0	0.93 (0.69, 1.24)	0.93 (0.64, 1.34)	0.48 (0.12, 1.94)	0.290
	**Caloric soft drinks**				
No. of cases	945	189	157	27	
Rate per 10,000 person-years	8.2	6.8	6.0	4.8	
Age-adjusted HR (95% CI)	1.0	0.96 (0.82, 1.12)	1.07 (0.90, 1.27)	1.08 (0.74, 1.59)	
Multivariable-adjusted HR (95% CI)					
Model 1^ǂ^	1.0	0.96 (0.82, 1.12)	1.04 (0.88, 1.24)	1.03 (0.70, 1.51)	
Model 2^¥^	1.0	0.97 (0.82, 1.14)	1.05 (0.88, 1.25)	0.95 (0.64, 1.43)	
Final model^¤^	1.0	0.98 (0.83, 1.15)	1.07 (0.89, 1.28)	0.98 (0.65, 1.46)	0.730

*Total person-time: 1,743.453 years.

^†^ 1 serving of caloric soft drink is 12 fluid ounces, 1 serving of sweetened bottled water/tea or fruit drink is 8 fluid ounces. Models were reciprocally adjusted for the other sugar-sweetened beverage types. HR, hazard ratio; CI, confidence interval.

^ǂ^Model 1 adjusted for: age, race/ethnicity, socioeconomic status, total years smoked, alcohol intake, colorectum cancer family history of first-degree relatives, history of polyps, diabetes, physical activity, aspirin use, multivitamin use, menopausal status, menopausal hormone therapy use, oral contraceptive use.

^¥^Model 2 adjusted for: Model 1 and body mass index, total energy intake, and dietary variables: red meat, processed meat, and vegetable intakes.

^¤^Final model adjusted for: age, total years smoked, alcohol intake, colorectum cancer family history of first-degree relatives, history of polyps, multivitamin use, menopausal status, menopausal hormone therapy use, body mass index, and total energy intake.

Sensitivity analyses excluding events diagnosed during the first 2 and 4 years after baseline did not change the direction or significance of the association between SSB consumption and risk of CRC (total, proximal colon cancer, and distal colorectum cancer) (S2 and S3 Tables). Also, as noted in the Methods, we assessed SSB intake according to BMI categories to assess the biological hypothesis of a stronger association of SSB and CRC risk in overweight/obese women. We found no evidence in support of this hypothesis (HR [95% CI] for total CRC risk among women with BMI ≥ 25 kg/m^2^, was 0.94 (0.76, 1.18) comparing daily versus rarely/never consumers of SSBs).

## Discussion

Results of this study showed no significant association between SSB consumption and total CRC, proximal colon cancer, and distal colorectum cancer. This was consistent regardless of covariate adjustment and remained after removal of CRC cases 2 and 4 years after baseline. There was no significant association between specific type of SSB and risk of total CRC.

It is challenging to compare our findings with those of the existing literature, since the assessment of this exposure and outcome association has been limited. Initial published literature on this topic focused on caloric soft drink consumption and colon cancer risks. A 2010 meta-analysis [30] pooling primary data from 10 cohorts reported a null association between intake of sugar-sweetened carbonated soft drinks and incident colon cancer (pooled multivariable relative risk [RR] = 0.94 [95% CI 0.66, 1.32]; *P* for trend = 0.91), among those consuming >550 grams/day (approximately 18 fluid ounces) versus non-consumers [30].

More recent studies have included broader exposures and endpoints and are consistent with our findings. In a study of adult women, Fung et al. [36] reported no association between SSB intake and risk of CRC (RR = 1.04 (95% CI 0.94, 1.16 for each serving/day increase of SSB), which is consistent with results from a cohort study of French adults (HR = 1.08 [95% CI 0.72, 1.71] for every 100 mL/day increase in SSB intake) [37]. Results from the Melbourne Collaborative Cohort Study [29] showed a higher risk of CRC (HR = 1.28 [95% CI 1.04, 1.57]) in individuals consuming ≥1 soft drink/day versus those who never consume these [29]. Our results show associations in the same direction as those of the Melbourne study, but did not reach statistical significance.

Biological mechanisms for a SSB association with colon or CRC risk have been proposed, including that described by Giovannucci [38], which might help explain our results and those in the literature. This framework elucidates the inter-relationship and synergy between dietary pattern (versus a single nutrient and/or food), physical activity, and weight status, and how these elements stimulate and/or inhibit hormonal functioning and inflammation and their impact on cancer risk. The insulin/insulin-like growth factor 1 (IGF-1) dyad is considered a key player in the activation and/or regulation of crucial pathways by which mitosis and apoptosis ensue [39]. Hence, hyperinsulinemia and elevated bioavailable IGF-1 levels support a carcinogenic and early tumor growth setting in some cancers, such as in the case of CRC, where this association has been determined, independent of adiposity [35], [40], [41]. Thus, if insulin is a marker for the causal factor of CRC risk, the entire dietary pattern influencing insulin levels may be expected to be associated with risk of CRC. Indeed, an empirical insulinemic dietary pattern formed in the Nurses’ Health Study and Health Professionals Follow-Up Study was associated with about a 30% increased risk of CRC (as well as other digestive system cancers) in these cohorts [42]. Although SSB did contribute to this dietary pattern, it was only one of 18 items, and the full dietary pattern was high in animal products, refined starches, sugars, and SSBs, while lower in whole grains, whole fruits, and green leafy vegetables [43]. Given that the insulinemic dietary pattern yielded a relative risk of about 1.3 for CRC, a single factor such as SSB should yield a relative risk substantially lower than 1.3, assuming insulinemia mediated (or acted as a marker) for the entire effect of SSB on CRC risk. In this context, the modest, non-significant HRs we observed are compatible with this hypothesis.

Our study has several strengths. A large analytic sample allowed us to conduct subgroup analyses by anatomical location; its prospective design addressed recall bias; and due to linkage with SEER cancer registry for endpoint ascertainment, we had a high follow-up rate (>99%) as applying to high follow-up for cancer endpoints (i.e., rather than for all participants). Sensitivity analysis addressed possibility of reverse causality, yet the number of CRC cases was reduced, and affected statistical power. In spite of these strengths, because only a small percentage (4.3%) of participants reported high SSB consumption (≥1 serving/day), we had limited power to assess high intake or dose-response. Also, although we presented beverage-specific analysis, the interpretation of such findings is limited due to inadequate sample size; there were few cases of total of CRC, especially in women consuming ≥1 serving/day of fruit drinks and caloric soft drinks. Our use of a single dietary assessment at baseline introduces the possibility of measurement error in assessing long-term intake if consumption patterns changed during follow-up. We were also limited to only a single estimate of SSB intake assessed at baseline, thereby we acknowledge the possibility of random measurement error. In addition, we cannot rule out that participants may have changed their beverage consumption intake and changes over time. SSB consumption trends among U.S. adults has declined in recent years [44], [45], thus in comparison to our findings, we would expect an attenuation in the magnitude of the measure of association with current tendencies. Finally, our study population was female and primarily non-Hispanic white, thereby limiting the generalizability of our results to other populations.

## Conclusion

In conclusion, we observed a non-significant association between SSB consumption and CRC risk. Given the biologic effects of SSBs on metabolic pathways that are important in colon and rectal cancer etiology, continued investigation is warranted to fully understand these important dietary exposures. Additionally, we propose that future studies repeatedly measure SSBs and address changes in consumption over time. We also encourage adequately powered cohorts to examine the association between SSBs and CRC risk, and, if possible, address racial/ethnic and gender-specific differences.

## Supporting information

S1 Table Comprehensive baseline characteristics of California Teachers Study participants according to sugar-sweetened beverage consumption categories*. (DOCX)

S2 Table Colorectal cancer risk* according to sugar-sweetened beverage consumption after removal of events that occurred at 2 years follow-up (n = 97,776). (DOCX)

S3 Table Colorectal cancer risk* according to sugar-sweetened beverage consumption after removal of events that occurred at 4 years follow-up (n = 95,667). (DOCX)
