# Supplementary material for: Sugar-sweetened beverages and colorectal cancer risk in the California Teachers Study
Source: PLoS One. 2019 Oct 9;14(10):e0223638. doi: 10.1371/journal.pone.0223638 (PMC6785057; doi:10.1371/journal.pone.0223638)
Supplement: S1 Table — (DOCX) [file pone.0223638.s001.docx]

**S1 Table.** Comprehensive Baseline Characteristics of California Teachers Study Participants According to Sugar-Sweetened Beverage Consumption Categories^*^

| Characteristic | Rare or  never | >rare/never to <1 serving per week | ≥1 serving per week to <1 serving per day | ≥1 serving  per day |
| --- | --- | --- | --- | --- |
| **N** | 40,911 | 33,198 | 21,403 | 4,286 |
| **Age, y** | 55.4 ± 0.06 | 48.9 ± 0.07 | 48.8 ± 0.09 | 48.8 ± 0.20 |
| **Race/ethnicity, %** |  |  |  |  |
| Asian/PI | 1,157 (2.8) | 1,512 (4.6) | 824 (3.9) | 118 (2.8) |
| African-American | 729 (1.8) | 1,113 (3.4) | 688 (3.2) | 117 (2.7) |
| Hispanic/Latino | 1,309 (3.2) | 1,736 (5.2) | 1,109 (5.2) | 192 (4.5) |
| Native American | 316 (0.8) | 234 (0.7) | 159 (0.7) | 26 (0.6) |
| White | 36,667 (89.6) | 27,919 (84.1) | 18,147 (84.8) | 3,750 (87.5) |
| Other/Mixed | 428 (1.1) | 450 (1.4) | 302 (1.4) | 55 (1.3) |
| Unknown | 305 (0.8) | 234 (0.7) | 188 (0.9) | 28 (0.7) |
| **Education, % ǂ** |  |  |  |  |
| Academic doctorate | 795 (1.9) | 557 (1.7) | 365 (1.7) | 94 (2.2) |
| Professional doctorate | 217 (0.5) | 162 (0.5) | 129 (0.6) | 26 (0.6) |
| Master’s degree | 10,494 (25.7) | 8,934 (26.9) | 5,686 (26.6) | 1,150 (26.8) |
| Bachelor’s degree | 9,112 (22.3) | 7,746 (23.3) | 4,518 (21.1) | 868 (20.3) |
| Associate’s degree | 130 (0.3) | 132 (0.4) | 92 (0.4) | 17 (0.4) |
| Technical school/certificate/High school | 8 (0.0) | 6 (0.0) | 8 (0.0) | 4 (0.1) |
| Less than High school | 0 | 1 (0) | 0 | 0 |
| Unknown | 20,155 (49.3) | 15,660 (47.2) | 10,605 (49.6) | 2,127 (49.6) |
| **Occupation, %** |  |  |  |  |
| Teacher, single grade Pre-K to High school | 17,994 (44.0) | 18,551 (55.9) | 12,149 (56.8) | 2,475 (58.8) |
| Teacher, other | 2,885 (7.1) | 2,478 (7.5) | 1,636 (7.6) | 364 (8.5) |
| Multiple | 193 (0.5) | 251 (0.8) | 147 (0.7) | 30 (0.7) |
| Pupil Services | 1,146 (2.8) | 1,099 (3.3) | 690 (3.2) | 136 (3.2) |
| Administration | 1,311 (3.2) | 1,221 (3.7) | 866 (4.1) | 198 (4.6) |
| Teacher, Pre-K/Elem/Other or JrH/Hi/Other | 270 (0.7) | 284 (0.9) | 183 (0.9) | 42 (1.0) |
| Pupil Services/Admin or  Pupil Services/Admin/Teacher | 400 (1.0) | 351 (1.1) | 241 (1.1) | 43 (1.0) |
| Unknown | 16,712 (40.9) | 8,963 (27.0) | 5,491 (25.7) | 1,080 (24.0) |
| **Socioeconomic status, %** |  |  |  |  |
| 1^st^ quartile, low | 1,614 (4.0) | 1,493 (4.5) | 959 (4.5) | 177 (4.1) |
| 2^nd^ quartile, low-medium | 6,728 (16.5) | 5,846 (17.6) | 3,835 (17.9) | 718 (16.8) |
| 3^rd^ quartile, medium-high | 13,030 (31.9) | 11,072 (33.4) | 6,891 (32.2) | 1,436 (33.5) |
| 4^th^ quartile, high | 19,017 (46.5) | 14,328 (43.2) | 9,435 (44.1) | 1,904 (44.4) |
| Unknown | 522 (1.3) | 459 (1.4) | 283 (1.3) | 51 (1.2) |
| **Marital status, %** |  |  |  |  |
| Married | 18,457 (45.1) | 16,268 (49.0) | 10,016 (46.8) | 1,973 (46.0) |
| Divorced | 3,547 (8.7) | 2,688 (8.1) | 1,637 (7.7) | 359 (8.4) |
| Separated | 284 (0.7) | 289 (0.9) | 188 (0.9) | 33 (0.8) |
| Widowed | 3,346 (8.2) | 1,558 (4.7) | 999 (4.7) | 178 (4.2) |
| Never married | 1,974 (4.8) | 1,685 (5.1) | 1,079 (5.0) | 2761(6.1) |
| Unknown | 13,303 (32.5) | 10,710 (32.3) | 7,484 (35.0) | 1,482 (34.6) |
| **Dietary Intake** |  |  |  |  |
| Energy, kcal/day | 1755.3 ± 3.35 | 1954.4 ± 3.72 | 2046.5 ± 4.64 | 2255.4 ± 10.36 |
| Carbohydrate, g/day | 251.5 ± 0.18 | 253.4 ± 0.20 | 260.2 ± 0.25 | 282.6 ± 0.55 |
| Protein, g/day | 80.6 ± 0.07 | 76.8 ± 0.07 | 74.4 ± 0.09 | 68.0 ± 0.20 |
| Total Fat, g/day | 60.0 ± 0.07 | 61.6 ± 0.07 | 59.8 ± 0.09 | 53.7 ± 0.20 |
| Fruit & Vegetables, g/day | 359.7 ± 0.87 | 299.9 ± 0.96 | 285.6 ± 1.19 | 266.1 ± 2.67 |
| Vegetables, g/day | 183.8 ± 0.54 | 163.4 ± 0.60 | 163.4 ± 0.75 | 167.7 ± 1.67 |
| Red meat, g/day | 29.4 ± 0.17 | 34.7 ± 0.19 | 36.1 ± 0.23 | 37.3 ± 0.52 |
| Processed meat intake, g/day | 6.5 ± 0.06 | 8.2 ± 0.06 | 8.5 ± 0.08 | 8.9 ± 0.18 |
| Total dairy intake, g/day | 224.1 ± 0.98 | 224.1 ± 1.09 | 215.6 ± 1.36 | 212.9 ± 3.04 |
| Dietary folate intake, g/day | 420.0 ± 0.81 | 436.4 ± 0.90 | 438.6 ± 1.12 | 442.5 ± 2.50 |
| **SSB intake, fl oz** | 0 ± 0.02 | 2.6 ± 0.02 | 5.5 ± 0.02 | 13.5 ± 0.05 |
| **MVPA, minutes/week** | 236.3 ± 1.22 | 213.7 ± 1.35 | 219.5 ± 1.68 | 220.7 ± 3.76 |
| **Smoking, %** |  |  |  |  |
| Never | 25,837 (63.2) | 23,304 (70.2) | 14,680 (68.6) | 2,785 (65.0) |
| Former | 12,942 (31.6) | 8,375 (25.2) | 5,597 (26.2) | 1,175 (27.4) |
| Current | 2,084 (5.1) | 1,494 (4.5) | 1,100 (5.1) | 321 (7.5) |
| Unknown | 48 (0.1) | 25 (0.1) | 26 (0.1) | 5 (0.1) |
| **Number of cigarettes per day, ¥** | 13.3 ± 0.08 | 11.5 ± 0.10 | 12.1 ± 0.13 | 14.4 ± 0.27 |

**S1 Table.** Comprehensive Baseline Characteristics of California Teachers Study Participants According to Sugar-Sweetened Beverage Consumption Categories^*^, Continued

| Characteristic | Rare or  never | | >rare/never to <1 serving per week | ≥1 serving per week to <1 serving per day | ≥1 serving  per day |
| --- | --- | --- | --- | --- | --- |
| **Total years smoked, ¥** | | 21.1 ± 0.11 | 17.8 ± 0.14 | 18.4 ± 0.17 | 20.0 ± 0.36 |
| **Alcohol consumption, %** |  | |  |  |  |
| None | 13,950 (34.1) | | 10,513 (31.7) | 6,983 (32.6) | 1,636 (38.2) |
| <20 g/day | 23,005 (56.2) | | 20,308 (61.2) | 12,818 (59.9) | 2,328 (54.3) |
| ≥20 g/day | 3,956 (9.7) | | 2,377 (7.2) | 1,602 (7.5) | 322 (7.5) |
| **Body mass index, kg/m^2^** | 24.9 ± 0.03 | | 24.6 ± 0.03 | 25.0 ± 0.04 | 25.7 ± 0.08 |
| **Body mass index, kg/m^2^** |  | |  |  |  |
| Underweight, <18.5 | 982 (2.4) | | 888 (2.7) | 543 (2.5) | 116 (2.7) |
| Normal, 18.5-24.9 | 22,710 (55.5) | | 19,468 (58.6) | 12,088 (56.5) | 2,203 (51.4) |
| Overweight, 25-29.9 | 10,052 (24.6) | | 7,619 (23.0) | 5,060 (23.6) | 1,054 (24.6) |
| Obese, ≥30 | 5,487 (13.4) | | 4,217 (12.7) | 3,079 (14.4) | 787 (18.4) |
| Unknown | 1,680 (4.1) | | 1,006 (3.0) | 633 (3.0) | 126 (2.9) |
| **Hypertension, %** | 7,842 (19.2) | | 4,288 (12.9) | 3,022 (14.1) | 673 (15.7) |
| **Diabetes, %** | 1,712 (4.2) | | 434 (1.3) | 344 (1.6) | 109 (2.5) |
| **Aspirin use, %** |  | |  |  |  |
| Daily | 3,656 (8.9) | | 1,736 (5.2) | 1,231 (5.8) | 294 (6.9) |
| Up to 6x/week | 5,770 (14.1) | | 4,806 (14.5) | 3,154 (14.7) | 684 (16.0) |
| Regular use, unknown frequency | 258 (0.6) | | 171 (0.5) | 145 (0.7) | 21 (0.5) |
| Not regularly taken | 30,652 (74.9) | | 26,075 (78.5) | 16,604 (77.6) | 3,233 (75.4) |
| Unknown use | 575 (1.4) | | 410 (1.2) | 269 (1.3) | 54 (1.3) |
| **Antihypertensive medication use, at least 1 medication, %** | | |  |  |  |
| Daily | 7,201 (17.6) | | 3,679 (11.1) | 2,622 (12.3) | 599 (14.0) |
| Up to 6x/week | 566 (1.4) | | 404 (1.2) | 267 (1.3) | 75 (1.8) |
| Regular use, unknown frequency | 525 (1.3) | | 292 (0.9) | 209 (1.0) | 39 (0.9) |
| Not regularly taken | 32,044 (78.3) | | 28,414 (85.6) | 18,036 (84.3) | 3,519 (82.1) |
| Unknown use | 575 (1.4) | | 409 (1.2) | 269 (1.3) | 54 (1.3) |
| **Multivitamin use, %** |  | |  |  |  |
| Daily | 16,355 (40.0) | | 10,578 (31.9) | 6,930 (32.4) | 1,492 (34.8) |
| Up to 6x/week | 5,928 (14.5) | | 6,748 (20.3) | 3,983 (18.6) | 659 (15.4) |
| Never | 6,559 (16.0) | | 5,252 (15.8) | 3,430 (16.0) | 695 (16.2) |
| Unknown use | 12,069 (29.5) | | 10,620 (32.0) | 7,060 (33.0) | 1,440 (33.6) |
| **Cancer family history, % ¤** | 22,250 (54.4) | | 17,160 (51.7) | 11,083 (51.8) | 2,257 (52.7) |
| **Colorectum cancer family history, % §** | 3,791 (9.3) | | 2,562 (7.7) | 1,711 (8.0) | 312 (7.3) |
| **Menopausal status and menopausal HT use, %** |  | |  |  |  |
| Premenopausal | 13,084 (32.0) | | 16,777 (50.5) | 10,722 (50.1) | 2,098 (49.0) |
| PP, no HT | 5,639 (13.8) | | 2,879 (8.7) | 1,913 (8.9) | 380 (8.9) |
| PP, past HT | 3,241 (7.9) | | 1,620 (4.9) | 1,038 (4.9) | 214 (5.0) |
| PP, current HT Estrogen | 6,287 (15.4) | | 3,567(10.7) | 2,257 (10.6) | 480 (11.2) |
| PP, current HT Estrogen & Progesterone | 7,128 (17.4) | | 4,335 (13.1) | 2,739 (12.8) | 510 (11.9) |
| All other | 5,532 (13.5) | | 4,020 (12.1) | 2,734 (12.8) | 604 (14.1) |
| **Oral contraceptive use, %** |  | |  |  |  |
| Current | 1,528 (3.9) | | 2,435 (7.6) | 1,520 (7.4) | 315 (7.6) |
| Past | 22,996 (58.4) | | 21,194 (65.8) | 13,860 (67.0) | 2,782 (67.5) |
| Never | 14,798 (37.6) | | 8,497 (26.4) | 5,262 (25.4) | 1,013 (24.6) |
| Unknown if current or past | 71 (0.2) | | 72 (0.2) | 47 (0.2) | 12 (0.3) |

^*^Values are n (%) for categorical variables and means ± standard error of the means for continuous variables. ǂEducation was obtained after baseline, during fourth mail-in questionnaire follow-up, 2005-2006. ¥Former or current smokers. ¤Cancer family history includes breast, endometrial, ovarian, cervical, lung, thyroid, colon, rectal, prostate, melanoma, and skin cancers, and also leukemia, and Hodgkin’s lymphoma history, of first-degree relatives (parent, sibling, offspring). §Colorectum cancer family history includes disease in first-degree relatives (parent, sibling, offspring). Elem indicates Elementary; fl oz, fluid ounces; g/day, grams per day; Hi, High School; HT, hormone therapy; JrH, Junior High School; kcal/day, kilocalories per day; mo, months; MVPA, moderate-vigorous physical activity; PP, peri- or post-menopausal; Pre-K, pre-kindergarten; SSB, sugar-sweetened beverage; y, years.
