# Supplementary material for: Sugar-sweetened beverages and colorectal cancer risk in the California Teachers Study
Source: PLoS One. 2019 Oct 9;14(10):e0223638. doi: 10.1371/journal.pone.0223638 (PMC6785057; doi:10.1371/journal.pone.0223638)
Supplement: S2 Table — (DOCX) [file pone.0223638.s002.docx]

**S2 Table.** Colorectal Cancer Risk^*^ According to Sugar-Sweetened Beverage Consumption after removal of events that occurred at 2 years follow-up (n=97,776)

|  | **Sugar-Sweetened Beverage Consumption †** | | | |  |
| --- | --- | --- | --- | --- | --- |
| Colorectal Cancer | Rare or never | >rare/never to <1 serving per week | ≥1 serving per week to <1 serving per day | ≥1 serving  per day | P trend |
| **Total** |  |  |  |  |  |
| No. of cases | 596 | 330 | 232 | 50 |  |
| Rate per 10,000 person-years | 8.6 | 5.6 | 6.1 | 6.6 |  |
| Age-adjusted HR (95% CI) | 1.0 | 0.93 (0.82, 1.07) | 1.05 (0.90, 1.23) | 1.18 (0.88, 1.57) |  |
| Multivariable-adjusted HR (95% CI) | |  |  |  |  |
| Model 1^ǂ^ | 1.0 | 0.93 (0.81, 1.07) | 1.04 (0.89, 1.21) | 1.16 (0.87, 1.55) |  |
| Model 2^¥^ | 1.0 | 0.93 (0.81, 1.08) | 1.06 (0.91, 1.25) | 1.16 (0.86, 1.57) |  |
| Final Model^¤^ | 1.0 | 0.94 (0.82, 1.09) | 1.07 (0.91, 1.25) | 1.16 (0.86, 1.56) | 0.187 |
| **Proximal Colon** |  |  |  |  |  |
| No. of cases | 345 | 180 | 114 | 25 |  |
| Rate per 10,000 person-years | 5.0 | 3.0 | 3.0 | 3.3 |  |
| Age-adjusted HR (95% CI) | 1.0 | 0.94 (0.78, 1.13) | 0.96 (0.78, 1.19) | 1.12 (0.74, 1.68) |  |
| Multivariable-adjusted HR (95% CI) | |  |  |  |  |
| Model 1^ǂ^ | 1.0 | 0.95 (0.79, 1.14) | 0.96 (0.78, 1.20) | 1.11 (0.74, 1.67) |  |
| Model 2^¥^ | 1.0 | 0.97 (0.80, 1.17) | 0.98 (0.78, 1.23) | 1.11 (0.73, 1.69) |  |
| Final Model^¤^ | 1.0 | 0.95 (0.79, 1.15) | 0.97 (0.78, 1.21) | 1.11 (0.73, 1.68) | 0.799 |
| **Distal Colorectum** |  |  |  |  |  |
| No. of cases | 251 | 150 | 118 | 25 |  |
| Rate per 10,000 person-years | 3.6 | 2.5 | 3.1 | 3.3 |  |
| Age-adjusted HR (95% CI) | 1.0 | 0.93 (0.76, 1.14) | 1.16 (0.93, 1.45) | 1.25 (0.83, 1.89) |  |
| Multivariable-adjusted HR (95% CI) | |  |  |  |  |
| Model 1^ǂ^ | 1.0 | 0.91 (0.74, 1.12) | 1.13 (0.90, 1.41) | 1.22 (0.81, 1.85) |  |
| Model 2^¥^ | 1.0 | 0.90 (0.73, 1.12) | 1.17 (0.93, 1.47) | 1.23 (0.80, 1.89) |  |
| Final Model^¤^ | 1.0 | 0.93 (0.75, 1.15) | 1.19 (0.95, 1.49) | 1.22 (0.80, 1.86) | 0.098 |

*Total person-time: 1,741,103 years. † 1 serving of caloric soft drink is 12 fluid ounces, 1 serving of sweetened bottled water/tea or fruit drink is 8 fluid ounces. HR indicates hazard ratio; CI, confidence interval.

^ǂ^Model 1 adjusted for: age, race/ethnicity, socioeconomic status, total years smoked, alcohol intake, colorectum cancer family history of first-degree relatives, history of polyps, diabetes, physical activity, aspirin use, multivitamin use, menopausal status, menopausal hormone therapy use, oral contraceptive use.

^¥^Model 2 adjusted for: Model 1 and body mass index, total energy intake, and dietary variables: red meat, processed meat, and vegetable intakes.

^¤^Final model adjusted for: age, total smoke years, alcohol intake, colorectum cancer family history of first-degree relatives, history of polyps, multivitamin use, menopausal status, menopausal hormone therapy use, body mass index, and total energy intake.
