# Supplementary material for: Sugar-sweetened beverages and colorectal cancer risk in the California Teachers Study
Source: PLoS One. 2019 Oct 9;14(10):e0223638. doi: 10.1371/journal.pone.0223638 (PMC6785057; doi:10.1371/journal.pone.0223638)
Supplement: S3 Table — (DOCX) [file pone.0223638.s003.docx]

**S3 Table.** Colorectal Cancer Risk^*^ According to Sugar-Sweetened Beverage Consumption after removal of events that occurred at 4 years follow-up (n=95,667)

|  | **Sugar-Sweetened Beverage Consumption †** | | | |  |
| --- | --- | --- | --- | --- | --- |
|  | Rare or never | >rare/never to <1 serving per week | ≥1 serving per week to <1 serving per day | ≥1 serving  per day | P trend |
| **Colorectal Cancer** |  |  |  |  |  |
| No. of cases | 533 | 292 | 206 | 46 |  |
| Rate per 10,000 person-years | 7.7 | 5.0 | 5.5 | 6.1 |  |
| Age-adjusted HR (95% CI) | 1.0 | 0.91 (0.79, 1.05) | 1.03 (0.87, 1.21) | 1.19 (0.88, 1.61) |  |
| Multivariable-adjusted HR (95% CI) | |  |  |  |  |
| Model 1^ǂ^ | 1.0 | 0.91 (0.79, 1.05) | 1.02 (0.87, 1.20) | 1.17 (0.87, 1.59) |  |
| Model 2^¥^ | 1.0 | 0.92 (0.79, 1.07) | 1.06 (0.89, 1.26) | 1.20 (0.87, 1.64) |  |
| Final Model^¤^ | 1.0 | 0.92 (0.79, 1.07) | 1.05 (0.89, 1.25) | 1.17 (0.86, 1.59) | 0.215 |
| **Proximal Colon** |  |  |  |  |  |
| No. of cases | 317 | 164 | 103 | 23 |  |
| Rate per 10,000 person-years | 4.6 | 2.8 | 2.7 | 3.0 |  |
| Age-adjusted HR (95% CI) | 1.0 | 0.92 (0.76, 1.11) | 0.93 (0.74, 1.17) | 1.10 (0.72, 1.68) |  |
| Multivariable-adjusted HR (95% CI) | |  |  |  |  |
| Model 1^ǂ^ | 1.0 | 0.93 (0.76, 1.12) | 0.93 (0.75, 1.17) | 1.10 (0.72, 1.68) |  |
| Model 2^¥^ | 1.0 | 0.95 (0.78, 1.16) | 0.95 (0.75, 1.21) | 1.11 (0.71, 1.72) |  |
| Final Model^¤^ | 1.0 | 0.93 (0.77, 1.14) | 0.93 (0.74, 1.18) | 1.08 (0.70, 1.68) | 0.994 |
| **Distal Colorectum** |  |  |  |  |  |
| No. of cases | 216 | 128 | 103 | 23 |  |
| Rate per 10,000 person-years | 3.1 | 2.2 | 2.7 | 3.0 |  |
| Age-adjusted HR (95% CI) | 1.0 | 0.90 (0.72, 1.13) | 1.15 (0.91, 1.46) | 1.31 (0.85, 2.03) |  |
| Multivariable-adjusted HR (95% CI) | |  |  |  |  |
| Model 1^ǂ^ | 1.0 | 0.89 (0.71, 1.12) | 1.13 (0.89, 1.43) | 1.27 (0.83, 1.97) |  |
| Model 2^¥^ | 1.0 | 0.89 (0.71, 1.13) | 1.20 (0.93, 1.53) | 1.32 (0.84, 2.08) |  |
| Final Model^¤^ | 1.0 | 0.91 (0.73, 1.15) | 1.21 (0.95, 1.54) | 1.28 (0.82, 1.99) | 0.068 |

*Total person-time: 1,734,557 years. † 1 serving of caloric soft drink is 12 fluid ounces, 1 serving of sweetened bottled water/tea or fruit drink is 8 fluid ounces. HR indicates hazard ratio; CI, confidence interval.

^ǂ^Model 1 adjusted for: age, race/ethnicity, socioeconomic status, total years smoked, alcohol intake, colorectum cancer family history of first-degree relatives, history of polyps, diabetes, physical activity, aspirin use, multivitamin use, menopausal status, menopausal hormone therapy use, oral contraceptive use.

^¥^Model 2 adjusted for: Model 1 and body mass index, total energy intake, and dietary variables: red meat, processed meat, and vegetable intakes.

^¤^Final model adjusted for: age, total smoke years, alcohol intake, colorectum cancer family history of first-degree relatives, history of polyps, multivitamin use, menopausal status, menopausal hormone therapy use, body mass index, and total energy intake.
